# Kinanthropometry and dietary habits of non-professional rugby players

**DOI:** 10.3389/fspor.2024.1439358

**Published:** 2024-07-08

**Authors:** Francis E. Holway, Francesco Campa, Cristian Petri, Luciano R. Spena, Natalia Y. Szydlowski

**Affiliations:** ^1^ Departamento de Medicina Aplicada a los Deportes, Club Atlético River Plate, Buenos Aires, Argentina; ^2^ Department of Biomedical Sciences, University of Padua, Padova, Italy; ^3^ Department of Sport and Informatics, Section of Physical Education and Sport, Pablo de Olavide University, Sevilla, Spain; ^4^ Medical Department of A.C.F. Fiorentina S.r.l., Florence, Italy; ^5^ Departamento de Nutrición, Universidad de Morón, Buenos Aires, Argentina; ^6^ Departamento de Medicina, CEFAR, Buenos Aires, Argentina

**Keywords:** body composition, anthropometry, nutrition, sports team, multicomponent models

## Abstract

**Introduction:**

Evaluating the body composition and dietary habits of non-professional athletes can help identify areas for improvement to enhance sports performance. The present study aimed to describe the anthropometric and body composition features, as well as the dietary habits, of non-professional rugby players in Argentina.

**Methods:**

Fifty-seven rugby players from a Group III Club of the Unión de Rugby de Buenos Aires (URBA) were assessed using extensive anthropometric measurements according to the International Society for the Advancement of Kinanthropometry (ISAK) protocol. Reference data from professional rugby players in Group I clubs were used as a control for body composition comparisons. Dietary intake was evaluated using the 24-h recall method, and nutrient analysis was performed with SARA software.

**Results:**

Non-professional rugby players were shorter (Forwards: 175.9 vs. 181.5 cm; Backs: 172.5 vs. 175.7 cm), had higher body fat percentages (Forwards: 16.4 vs. 12.3%; Backs: 11.0 vs. 9.3%), and were less muscular (Forwards: 46.0 vs. 48.8%; Backs: 48.4 vs. 50.2%) compared to professional rugby players. The average dietary intake was 3,363 Kcal, with protein and carbohydrate intakes of 1.4 g kg^−1^ day^−1^ and 4.1 g kg^−1^ day^−1^, respectively, and 35% of energy intake from fat. Backs reported a higher caloric intake than forwards (3,682 vs. 2,827 Kcal). There was a high prevalence of insufficient intake of calcium (58%), vitamin A (49%), and vitamin C (65%), the latter two corresponding with a low intake of fruits and vegetables (6% of total energy intake). Meal pattern analysis showed that 46% of total energy was ingested at dinner.

**Conclusions:**

The body composition of non-professional rugby players from low-income clubs could be improved to enhance rugby performance, as compared to players in more competitive tiers. Economic constraints might contribute to a sub-optimal nutritional profile, potentially affecting body composition and on-field performance negatively. Recommendations to improve dietary intake should be made considering the budget constraints of these players.

## 1 Introduction

**Figure 1:**
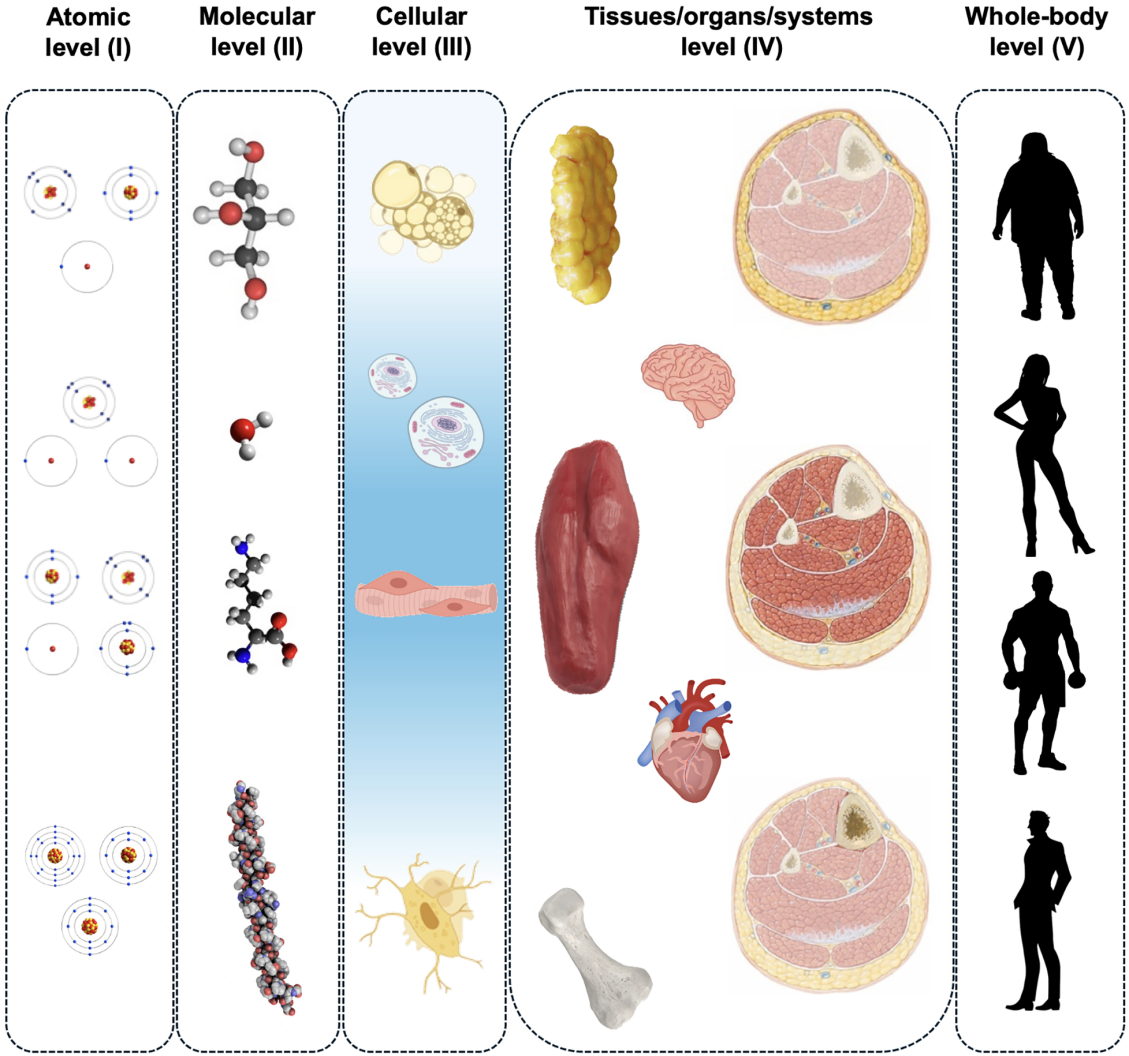
The five levels of body composition.

Body composition describes the various components that make up body mass. These components can be described and organized according to increasing levels of complexity, divided into five distinct levels (1), as shown in Figure 1. For example, an oxygen atom combined with two hydrogen atoms forms a water molecule, which is then incorporated into different cells, albeit in varying quantities, as well as into extracellular spaces. Different groups of cells are then organized to form tissues, which generate organs and result in systems that are part of the fourth level of organization. Regarding this fourth level, each component results from the sum of different types of molecules. For instance, visceral and internal adipose tissue not only include non-essential lipids that constitute fat mass at the molecular level but also small amounts of water, proteins, and other molecules that combine to form vessels and connective components, which are part of the adipose tissue (2).

The quantification of body composition parameters can be achieved through indirect methods or using predictive formulas based on anthropometric or bioimpedance measurements and is generally tailored to the specific needs of the groups being assessed. In sports, it is common to consider parameters such as fat mass, body water, adipose tissue, and muscle tissue. Additionally, raw parameters based on anthropometric measurements can be used in a qualitative approach by assessing somatotype components or simply comparing them with reference values using *z*-scores (3).

Rugby is a high-intensity intermittent contact sport that relies on glycogen as a principal fuel source and requires a moderate-to-high carbohydrate intake (7–8 g kg^−1^ day^−1^) to replenish stores (4, 5). Additionally, the evolution of rugby players' physiques, especially since the adoption of professionalism in 1995, necessitates dietary and exercise intervention programs to increase size and muscle mass and reduce body fat (6). The dimensional characteristics of players can result by selection pressures, with lower competitive gradient leagues presenting smaller players (7). Different playing positions in rugby can also lead to variations in the size and body composition of the players. For instance, forwards, who are often involved in physical confrontations and scrums, tend to have greater body mass and higher fat percentages compared to backs, who generally require more speed and agility and thus have leaner and more muscular physiques (8).

The nutritional recommendations for increasing muscle mass include consuming 1.2–1.6 g kg^−1^ day^−1^ of protein, along with sufficient energy and carbohydrate intake to support growth. These dietary guidelines should be combined with proper resistance training programs to maximize muscle hypertrophy (9). To optimize muscle growth, it is recommended that moderate protein intake should come from high-quality sources such as milk, eggs, and meat (10). Although health concerns regarding fat, saturated fat, and cholesterol intake have been highlighted in American football and rugby league, the primary focus of nutrition for athletes has been on performance. Consequently, the recommended total fat intake for athletes is between 20% and 25% of their energy intake (5).

Sport-specific nutrition recommendations may be influenced by socio-economic factors affecting food choices. Non-professional athletes with limited budgets might opt for high-energy density foods, particularly in low socio-economic status areas of countries like Argentina, where rugby has not yet fully embraced professionalism (11, 12). Despite the worldwide popularity of rugby, there is a lack of studies pertaining to the dietary intake of these athletes (13, 14).

Therefore, the purpose of this study is to present the dietary intake and anthropometric profile of non-professional rugby players in Argentina. This research aims to fill the gap in dietary information on this sport and highlight how low budgets influence food selection and nutrient composition.

## 2 Materials and methods

### 2.1 Subjects

A battery of anthropometric variables (body mass, height, sitting height, bone breadths, limb and trunk girths, and skinfolds) was collected on 57 rugby union players from a Group III Club of the Unión de Rugby de Buenos Aires (URBA) set in a low-income neighborhood. Measurements were performed by level 2 and 3 anthropometrists who adhered to the International Society for the Advancement of Kinanthropometry (ISAK) protocol (15).

### 2.2 Anthropometry

Anthropometric tools included an Aspen EB6571 digital strain-gauge scale (Jinli Electronic, Zhongshan, China), wall-mounted portable stadiometers with headboards (Rosscraft SRL, Argentina), a 50-cm sturdy wooden box for sitting height, bone breadth calipers (Rosscraft SRL, Argentina), measuring tapes (Rosscraft, Canada), and Holtain skinfold calipers (Crymych, UK). Body composition was assessed using the Five-Way Fractionation Method (16) which divided the body into anatomically defined tissue masses: adipose, muscle, residual, bone, and skin. Fat mass was estimated using the Yuhasz percent fat equation (17). Body mass index (BMI), weight/height^2^, sum of six skinfolds (S6skf) as the sum in millimeters of triceps, subscapular, supra-spinale, abdominal, front thigh and medial calf skinfolds, and muscle-to-bone ratio (MBR) as muscle mass/skeletal mass (8) were calculated. Registered dietitians interviewed the players to record a 24-hour dietary recall of the previous day's (a Friday) food and beverage intake, using memory cues and strategies to optimize recall. Diet analysis (energy, macro-, micro-nutrients, meal, and food-type energy distribution) was carried out using the SARA nutrition software (version 1.2.12, Ministerio de Salud, Argentina; http://www.msal.gov.ar/htm/Site/ennys/site/sara.asp). Micro-nutrient adequacy was established by comparing the reported intakes with the Daily Reference Intakes (DRI) for adult males suggested by the Food and Nutrition Board, Institute of Medicine, USA (http://www.nap.edu/catalog/dri/). Additional information on weekly training and daily activities was collected to estimate a physical activity level (PAL) using the Factorial Method (Energy and protein requirements. Report of a joint FAO/WHO/UNU Expert Consultation., 1985). Total energy expenditure estimation (TEE) was calculated by multiplying the PAL by the estimated basal metabolic rate (BMR) using the Schofield equation (18). Validity of reported food intakes was assessed using the suggestions of Black (19), comparing reported energy intake (EI) against calculated TEE. To avoid over-estimating BMR in over-fat players, the Hamwi formula was used to adjust the body weight used in calculating BMR (20). Before testing, the purpose of the study was explained to the players, who signed an informed consent form. Approval for the study was obtained from the Ethics Board at the Departamento de Medicina at River Plate Club.

### 2.3 Statistical analysis

The SPSS software (v27.0.0.0, SPSS Inc., Chicago, USA) was used for all statistical analyses. Data were presented as the mean ± standard deviation (SD). Assumptions of normality were verified using the Shapiro-Wilk test and student's independent *t*-test and Mann-Whitney *U*-test were used for comparison analyses. For all analyses, the criterion for significance was set at an alpha level of *p* < 0.05.

## 3 Results

The participants trained for two hours on Tuesdays and Thursdays and played games on Saturdays. They began playing rugby at an average age of 15.9 ± 5.2 years, with an average rugby-playing history of 11.7 ± 7.4 years. PAL was 1.83 ± 0.23.

**Figure 2:**
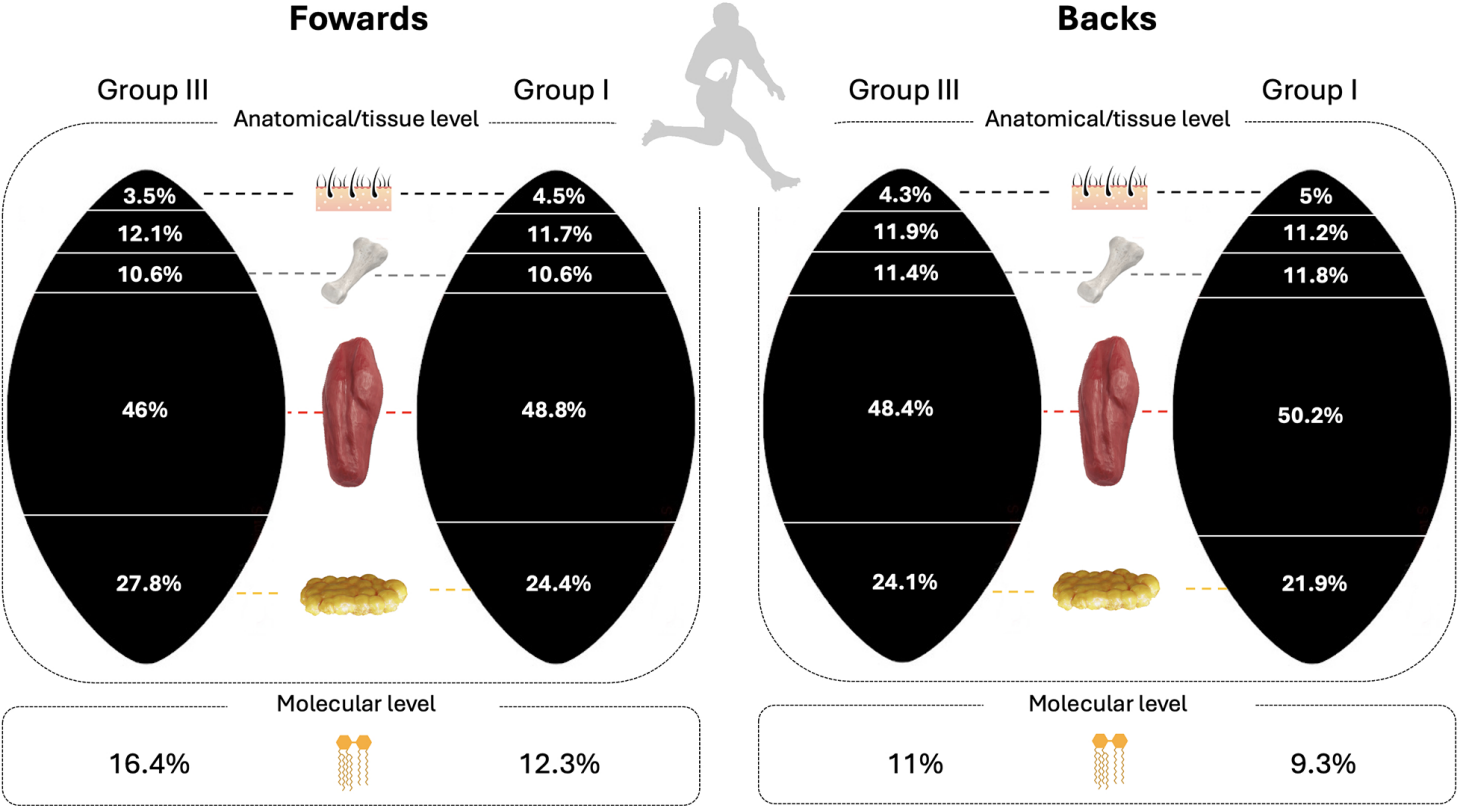
Body composition characteristics of professional and non-professional rugby players grouped by roles.

Table 1 presents body composition characteristics and comparison analyses for Group III non-professional players (G3) against reference data of Group I players (G1) (8). Figure 2 schematizes the main body mass components for the two groups.

**Table 1:** Body composition features by position of group III non-professional players (G3) against professional group I reference players (G1). Data are mean ± standard deviation.

Variable	Forwards				Backs			
	G3 *n* = 31	G1 *n* = 70	Dif.	*p*-value	G3 *n* = 26	G1 *n* = 63	Dif.	*p*-value
Age (years)	28.2 ± 6.8	24.5 ± 3.5	3.7	0.007*	26.4 ± 8.1	24.2 ± 3.6	2.2	0.198
Weight (kg)	100.3 ± 16.6	98.1 ± 10.7	2.1	0.516	78.9 ± 6.5	79.8 ± 8.2	−0.9	0.612
Height (cm)	175.9 ± 4.8	181.5 ± 6.8	−5.7	0.000*	172.5 ± 5.2	175.7 ± 6.7	−3.2	0.031*
% adipose tissue	27.8 ± 4.6	24.4 ± 3.4	3.4	0.000*	24.1 ± 4.7	21.9 ± 3.0	2.2	0.036*
% muscle mass	46.0 ± 3.9	48.8 ± 2.9	−2.8	0.000*	48.4 ± 4.2	50.2 ± 2.4	−1.8	0.049*
% residual	12.1 ± 1.0	11.7 ± 0.7	0.4	0.069	11.9 ± 0.9	11.8 ± 0.8	0.2	0.381
% skeletal mass	10.6 ± 0.9	10.6 ± 0.9	0.0	0.876	11.4 ± 0.9	11.2 ± 1.0	0.1	0.635
% skin	3.5 ± 0.5	4.5 ± 0.3	−1.0	0.000*	4.3 ± 0.4	5.0 ± 0.3	−0.7	0.000*
Kg adipose tissue	28.4 ± 8.5	24.1 ± 5.0	4.3	0.013*	19.0 ± 4.1	17.5 ± 3.4	1.5	0.077
Kg muscle mass	45.8 ± 6.1	47.8 ± 5.3	−2.1	0.083	38.2 ± 4.4	40.0 ± 4.4	−1.9	0.071
Kg residual	12.2 ± 2.6	11.5 ± 1.5	0.7	0.186	9.4 ± 1.1	9.4 ± 1.1	0.0	0.924
Kg skeletal mass	10.6 ± 1.5	10.3 ± 1.2	0.2	0.428	8.9 ± 0.8	9.0 ± 1.0	0.0	0.967
Kg skin	3.4 ± 0.1	4.4 ± 0.3	−1.0	0.000*	3.4 ± 0.2	3.9 ± 0.3	−0.6	0.000*
BMI Kg/m^2^	32.4 ± 5.1	29.8 ± 3.3	2.6	0.012*	26.5 ± 2.1	25.8 ± 2.0	0.7	0.134
S6skf mm	131.1 ± 52.7	92.9 ± 28.8	38.3	0.001*	79.6 ± 26.5	63.5 ± 19.1	16.1	0.008*
MBR	4.3 ± 0.4	4.7 ± 0.5	−0.3	0.004*	4.3 ± 0.5	4.5 ± 0.5	−0.2	0.058
% Fat Yuhasz	16.4 ± 5.5	12.3 ± 3.0	4.0	0.001*	11.0 ± 2.9	9.3 ± 2.0	1.7	0.008*

* Statistically significant difference (*p* < 0.05). BMI, body mass index; S6, sum of six; MBR, muscle-to-bone ratio.

Energy and macro-nutrient intake were reported in Table 2. Backs players presented a higher energy intake than forward players, resulting from higher macro-nutrients intake. Micro-nutrient intake was reported in Table 3.

**Table 2:** Energy and macro-nutrient intake.

	Forwards *n* = 31		Backs *n* = 26		Dif.	*p*-value	All players *n* = 57	
	Median	Range	Median	Range			Median	Range
Energy Kcal	2,827	1,220–6,228	3,682	2,104–7,982	−855	0.044*	3,363	1,220–7,982
Protein								
g	117	56–329	136	47–292	−19	0.249	119	47–329
%	16.0%	7%−43%	14%	7%−32%	1.7%	0.491	15%	7%−43%
g·kg ^−1^ ·day ^−1^	1.1	0.5–3.6	1.8	0.7–3.5	−0.7	0.002*	1.4	0.5–3.6
Carbohydrates								
g	342	107–821	401	177–828	−59	0.269	373	107–828
%	50.0%	27%−79%	46%	25%−62%	3.9%	0.089	48%	25%−79%
g·kg ^−1^ ·day ^−1^	3.3	1.1–8.0	5.3	2.3–10.0	−2.1	0.006*	4.1	1.1–10.0
Fats								
g	104	33–334	149	70–428	−45	0.012*	143	33–428
%	32.0%	14%−54%	39%	25%−62%	−6.4%	0.016*	35%	14%−62%
g·kg ^−1^ ·day ^−1^	1.0	0.3–3.7	1.9	1.0–4.8	−0.9	0.000*	1.5	0.3–4.8
Fiber g	17	8–38	17	6–41	0	0.328	17	6–41

* Statistically significant difference (*p* < 0.05).

**Table 3:** Micro-nutrient intake of non-professional rugby players showing adequacy and prevalence of insufficient and excessive intakes.

Micro-nutrient	Median	Range	% RDA^a^		Prev < EAR^b^		Prev > UL^c^	
			Median	95% C.I.	Cases	%	Cases	%
Minerals								
Iron mg	66	10–68	313%	37%	0	0%	4	7%
Sodium mg	2,526	4,856–14,069	168%	40%	9	16%	31	54%
Potassium mg	2,862	916–7,185	124%	18%	8	14%		
Calcium mg	738	199–2,435	74%	13%	33	58%	0	0%
Phosphorus mg	1,886	788–3,596	269%	26%	0	0%	0	0%
Zinc mg	19	7–42	169%	24%	10	18%	3	5%
Vitamins								
Niacin mg	38	11–94	239%	31%	1	2%	34	60%
Folate μg	777	129–2,555	194%	32%	7	12%	17	30%
Vitamin A μg	626	76–2,257	70%	13%	28	49%	0	0%
Thiamin mg	3.8	1.1–11.1	314%	42%	0	0%		
Riboflavin mg	3.1	0.6–19.3	241%	61%	2	4%		
Vitamin B12 μg	8.0	1.5–32.4	333%	71%	2	4%		
Vitamin C mg	51	0–458	56%	29%	37	65%	0	0%

^a^ RDA, recommended dietary allowance.

^b^ EAR, estimated average requirements.

^c^ UL, tolerable upper intake levels.

**Figure 3:**
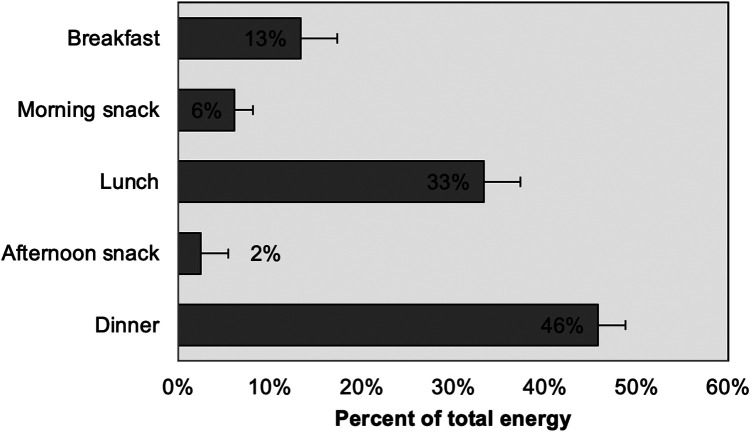
Average distribution of energy intake per meal. Error bars are 95% C.I.

Twenty-three percent of the players surveyed skipped breakfast, while another 37% consumed less than 10% of their daily energy intake during this meal. This means that 60% of the players had deficient breakfasts. Figure 3 illustrates the average distribution of energy intake per meal.

**Figure 4:**
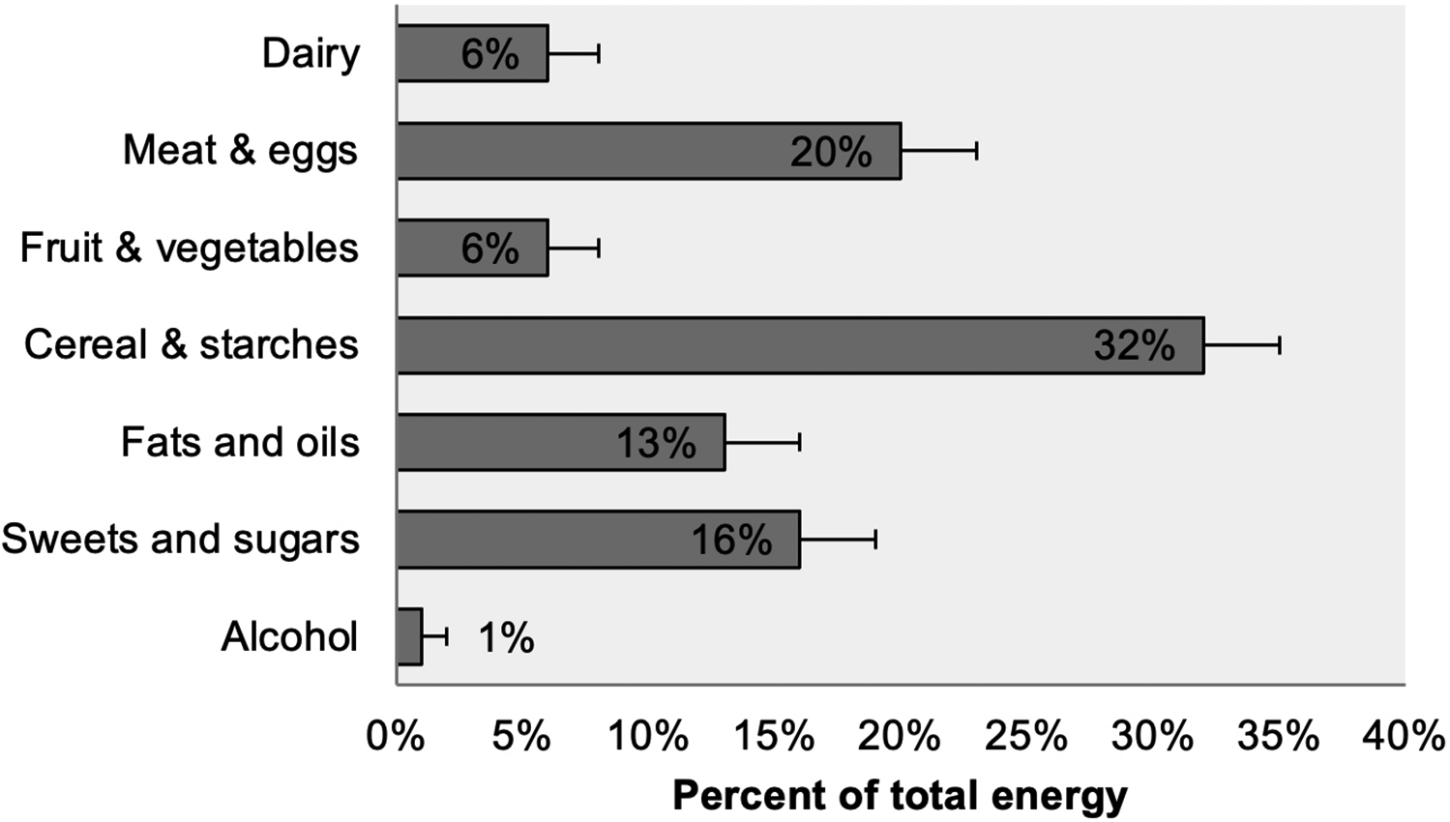
Average distribution of energy intake per type of food. Error bars are 95% C.I.

Five players reported to have drunk alcohol on the recorded day, with beer as the main option, followed by wine and spirits, as shown in Figure 4.

## 4 Discussion

The objective of the study was to present the anthropometric characteristics of non-professional rugby players along with their dietary habits. Understanding the characteristics of athletes included in low-income teams can help guide specific training or nutritional programs aimed at improving health status and sports performance.

### 4.1 Size and body composition

When compared to professional rugby players (Table 1), both forwards and backs from the non-professional team were shorter, had more adiposity, and possessed less muscle mass. These differences were anticipated due to the less competitive nature of their rugby environment, where such physical attributes are crucial for performance (7). The reasons for these disparities may include selection processes, living in disadvantaged socio-economic settings, genetics, the amount of training, and/or nutrition.

The use of the five-way fractionation model allowed for the quantification of important components such as muscle, bone, and adipose tissue. This enabled the calculation of the MBR, an index that can be particularly useful since muscle mass is correlated with strength and performance (21). Adipose tissue is defined as the anatomical entity, encompassing lipids, proteins, electrolytes, and water, and differs from chemically-defined fat mass which only refers to lipids (22). This explained why adiposity values were higher than percent fat values, as shown in Table 1. Considering previous findings, the fat mass calculated in the participants included in this study show resulted higher compared to elite rugby players as well as athletes from other sports teams (3, 23, 24).

### 4.2 Energy and macronutrients

Back players had a higher energy intake compared to the heavier forward players (Table 2). The median energy intake was 3,363 Kcal (range 1,220–7,982), which is lower than that of Australian rugby league players, whose median intake is 4,230 Kcal (range 2,671–6,917 Kcal) (13), presumably because the amateur players trained less. Since the PAL was not different between the two groups (Forwards 1.84, Backs 1.83), there may have been over-reporting of food intake by backs and/or under-reporting by forwards. When the ratio of energy intake to basal metabolic rate was derived, forwards had a ratio of 1.68 ± 0.67, which was less than the backs' ratio of 2.11 ± 0.63. These ratios suggest that the heavier forwards might have been underestimating their food intake, and backs overestimating it, assuming accurate food intake assessment would result in values similar to their PAL values.

Macronutrient intake (protein, carbohydrate, and fat) was higher in backs (1.8, 5.3, and 1.9 g kg^−1^ day^−1^, respectively) compared to forwards (1.1, 3.3, and 1.0 g kg^−1^ day^−1^, respectively). This discrepancy was expected since forwards were heavier and possibly under-reported their intake. The protein and carbohydrate intake for forwards were below the recommended values of 1.6 and 7–8 g kg^−1^ day^−1^. It may be worth considering that larger athletes might require smaller per-kilogram nutrient recommendations, or that some adjustment for lean mass could be necessary.

### 4.3 Micronutrient intake

The median values for the whole group exceeded the RDAs for all vitamins and minerals (Table 3), except for calcium (74%), vitamin A (70%), and vitamin C (56%). For these three micronutrients, the prevalence of players consuming less than the Estimated Average Requirement (EAR) was also the highest. Although the median calcium intake was 738 mg, which is not particularly low, the recommended daily allowance (RDA) of 1,000 mg is considered by some to be excessively high and is a topic of ongoing debate (25). The low intake of vitamins A and C, along with the low fiber intake, indicated a low intake of fruits and vegetables (Figure 4). The participants of this study reported a low intake of calcium and vitamin C than professional (13) and semi-professional rugby players (26) who reported consuming 1,400–1700 mg of calcium and 150–200 mg of vitamin C.

### 4.4 Energy intake per meal and type of food

Figure 3 showed that almost half of the daily energy intake was at dinner, while only 13% was at breakfast, with 23% of the participants used to skip breakfast. Training sessions for these players were schedule at night and were followed by dinner. This eating pattern may not optimize the balance between exercise and nutrition, as it involves insufficient intake before training and excessive consumption afterward. Although dietary periodicity studies are not very common, research including team sport athletes in Australia also found that these athletes tended to eat larger meals at night (27). Cereal and starches contributed most of the energy to the diets of these rugby players, followed by meat and eggs, sweets and sugars, and fats and oils (Figure 4). Less energy came from dairy products, and fruits and vegetables. Alcohol contributed 1% of energy intake, less than the 4%–5% reported by Lundy et al. (13), but this figure most likely underestimated real intake since the survey included a Friday before Saturday morning training. One of the impacts of a restricted budget on dietary choices was increasing the consumption of inexpensive starches, sugars, and oils, while diminishing that of higher-priced, nutrient-dense meats, dairy, fruits, and vegetables, as was found with these players, except for the high meat intake, which, although declining, is abundant in Argentina (28).

Some study limitations should be listed. For example, the 24-h dietary recall method had many pitfalls, including under-reporting by fatter individuals, and the software used for nutrient analysis might have had its own limitations; for instance, we were perplexed by the median value for iron intake, which at 66 mg was about two to three times larger than that data reported in previous studies (13, 26). Future studies should aim to obtain nutritional data from three-day weighted food records.

## 5 Conclusions

Rugby players from a low socio-economic area playing in a third-division level in Argentina were shorter, fatter, and had less muscle than their division one counterparts, and their diets were high in starches, sugars, and fats, and low in fruits and vegetables. To improve health, body composition, and sport-specific performance, this information might aid dietitians in suggesting food intake patterns and choices that improve this nutritional scenario within the budget constraints of the players.

## Data Availability

The raw data supporting the conclusions of this article will be made available by the authors, without undue reservation.
